# AutoFoci, an automated high-throughput foci detection approach for analyzing low-dose DNA double-strand break repair

**DOI:** 10.1038/s41598-018-35660-5

**Published:** 2018-11-23

**Authors:** Nicor Lengert, Johanna Mirsch, Ratna N. Weimer, Eik Schumann, Peter Haub, Barbara Drossel, Markus Löbrich

**Affiliations:** 1 Theory of Complex Systems, Darmstadt University of Technology, Hochschulstr. 6, 64289 Darmstadt, Germany; 2 Radiation Biology and DNA Repair, Darmstadt University of Technology, Schnittspahnstr. 13, 64287 Darmstadt, Germany; 3 Image Consulting, 68804 Altlußheim, Germany

## Abstract

Double-strand breaks (DSBs) are the most lethal DNA damages induced by ionising radiation (IR) and their efficient repair is crucial to limit genomic instability. The cellular DSB response after low IR doses is of particular interest but its examination requires the analysis of high cell numbers. Here, we present an automated DSB quantification method based on the analysis of γH2AX and 53BP1 foci as markers for DSBs. We establish a combination of object properties, combined in the object evaluation parameter (OEP), which correlates with manual object classification. Strikingly, OEP histograms show a bi-modal distribution with two maxima and a minimum in between, which correlates with the manually determined transition between background signals and foci. We used algorithms to detect the minimum, thus separating foci from background signals and automatically assessing DSB levels. To demonstrate the validity of this method, we analyzed over 600.000 cells to verify results of previous studies showing that DSBs induced by low doses are less efficiently repaired compared with DSBs induced by higher doses. Thus, the automated foci counting method, called AutoFoci, provides a valuable tool for high-throughput image analysis of thousands of cells which will prove useful for many biological screening approaches.

## Introduction

Every person is constantly exposed to different types of ionising radiation (IR) without even noticing the exposure. The mean radiation dose for people in Germany is about 4 mSv per year and encompasses the exposure from natural and man-made sources^1^. However, the individual exposure level can reach 20–50 mSv if medical examinations, such as computer tomography scans, are encountered^2,3^. IR induces a variety of different lesions, of which DNA double-strand breaks (DSBs) are considered to be biologically the most hazardous since unrepaired or misrepaired DSBs can lead to genomic instability and carcinogenesis^4–6^. The risk associated with exposure to low doses of radiation is subject of intense and highly controversial discussions, highlighting the necessity of studies investigating the effects of low radiation doses^7–11^.

Over the last years, many studies provided insight into new DSB repair factors or even new DSB repair pathways and therefore contributed to a better understanding of the cellular radiation response^12–15^. However, these studies were typically performed at high doses, while only a few studies addressed the radiation response after low radiation doses^16–23^, largely because the observed effects are often too small for a reliable assessment by standard biological methods. γH2AX foci analysis by immunofluorescence (IF) microscopy represents a method with sufficient sensitivity to detect DSBs after X-ray doses of 1-2 mGy (which is equivalent to 1-2 mSv for X-rays)^18,20^. γH2AX foci arise from the phosphorylation of the histone variant H2AX at a DSB site^24^ and can be visualized microscopically by using specific antibodies coupled to a fluorescence dye. They form within minutes after DSB induction and their loss reflects the completion of the repair process^20,25–27^. Surprisingly, the kinetics for the loss of γH2AX foci depend on the applied radiation dose and proceed more slowly after doses in the mGy range^17,18,20,23^. This impaired DSB repair efficiency at low radiation doses was observed in cultured human cells^18,20,23^ and verified by *in vivo* studies with mice^17,18^.

Since inefficient DSB repair processes may have an impact on the risk of low-dose exposures, further investigations on this effect are important. However, due to the small number of induced DSBs, low-dose studies are very time-consuming because they require the analysis of a high number of cells. While the evaluation process could be optimized by an automated foci counting approach, existing methods^28–33^ do not meet the requirements in the low-dose range regarding time efficiency, differentiation between foci and background signals, easy integration into standard equipment and reproducibility between different experiments (own experience and ref.^34^). The latter aspect is particularly crucial since independent experiments often show slightly different staining qualities with varying degrees of unspecific background signals. To discriminate foci from background signals, programs use either a fixed or an adjustable threshold. However, a fixed threshold does not adequately consider differences in staining quality while procedures for threshold adjustment are often arbitrary (see Supplementary Information).

Here, we present our automated high-throughput software tool “AutoFoci” to count γH2AX and 53BP1 foci in low-dose irradiated cells. AutoFoci detects objects that include foci and unspecific background signals and records various object properties such as intensity, size and sharpness. A combination of object properties correlates with a manual classification and shows a bi-modal distribution that can be used to discriminate foci from background signals. A manual adjustment step involving a small subset of critical objects allows the correction for different staining qualities. We applied AutoFoci to analyze more than 600,000 cells irradiated with doses between 12 mGy and 1 Gy and reproduced the impaired DSB repair efficiency after low radiation doses that was previously observed using manual foci counting^18,20^. Therefore, AutoFoci provides a unique tool to assess DSB repair processes after low doses of IR when many cells need to be analyzed.

## Results

### Experimental design, image acquisition and processing

**Figure 1:**
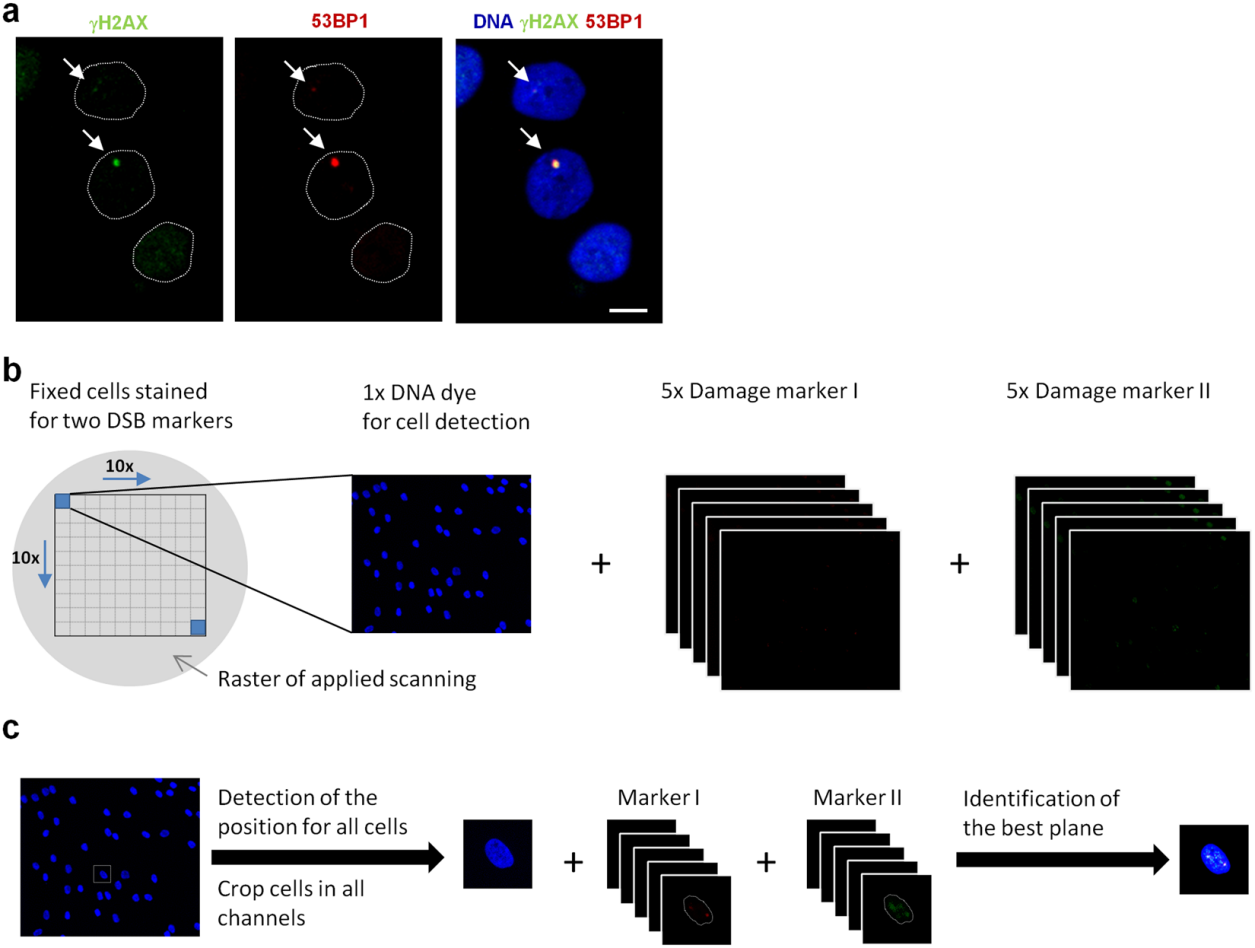
Generation of single cell images for automated foci quantification. Non-dividing HOMSF1 cells were fixed and stained for two DSB markers and DAPI. (**a**) IF images of the DSB markers γH2AX and 53BP1. Dotted lines indicate the shape of the nuclei in the IF images. The scale bar represents 10 µm. (**b**) Using a scanning microscope, a scan raster of 10 × 10 fields was applied and 1 DAPI image and z-stacks of 5 images for each DSB damage marker were captured with a step size of 1.2 µm around the focal plane. (**c**) Image-stacks were processed in several steps by using the Cellect tool. First, the positions of all cells were detected automatically by the DAPI signal. The position information was then used to crop cells in all channels to create single cell images. Second, the best image plane out of the 5 images for each damage marker was identified by maximal contrast.

The DNA damage response after exposure to low doses of IR is difficult to examine due to the small effects induced. A reliable analysis requires a suitable cell system with a low level of spontaneously occurring DSBs and a highly sensitive, high-throughput assay to measure DSB repair in several thousands of cells. To minimize the number of spontaneous breaks, we used HOMSF1 human fibroblasts that were maintained in confluence for at least two weeks to reduce DSB formation associated with DNA replication (Fig. S1a,b). We measured DSBs by the IF detection of 53BP1 and γH2AX foci which co-localize in non-dividing G0/G1-phase cells (Fig. 1a). The identification of DSBs by two independent markers optimizes DSB detection in the presence of unspecific background staining. The level of spontaneous DSBs in unirradiated cells assessed manually ranges from 0.15 to 0.3 foci per cell (Fig. S1c), corresponding to the number of DSBs induced by a dose of 7–15 mGy (Fig. S1d).

To establish a highly sensitive, high-throughput assay, we used a scanning fluorescence microscope equipped with an autofocus function to monitor 10 × 10 fields, each containing about 50 cells (Fig. 1b). For each field, the µManager software (Vale lab, UCSF) obtains one image of the blue channel to detect the cells via DAPI staining (a DNA staining dye) and z-stacks consisting of five images for each of the two DNA damage markers (Fig. 1b). The software tool Cellect identifies individual cell nuclei from the DAPI image and crops them to obtain single-cell images. It then selects from the 5 z-stack images the one with the highest contrast for further analysis (Fig. 1c). The image with the highest contrast best displays an identified focus while reducing the impact of unspecific background signals compared with a maximum intensity projection of all 5 planes. Finally, we included a step to identify any remaining S- and G2-phase cells and any potentially dying cells by their abnormal DAPI content, size or shape and excluded them from the analysis. All image acquisition and processing steps are performed automatically.

### Automated foci evaluation software “AutoFoci”

In order to automate the counting of co-localizing γH2AX and 53BP1 foci, we applied a three-step approach. First, an algorithm identifies objects within the cell nucleus, background signals as well as foci, and extracts all relevant object properties. The second step defines an object evaluation parameter (OEP) as a single measure that best correlates with the manual object rating. Finally, algorithms determine a threshold for the OEP to separate foci from background signals. This third step includes a manual adjustment of the threshold for each sample by rating a small set of objects at the automatically estimated threshold.

#### Object detection

Objects detected with our procedure include IR-induced and spontaneously occurring DSBs with robust foci signals as well as unspecific background signals resulting from staining artefacts. We set our object detection criteria such that the background signals outnumber the robust foci signals by a factor of 10 to 20. An algorithm detects objects in the red channel with the 53BP1 signal by the identification of local maxima which we defined as pixels with an intensity value higher than any surrounding pixel in a given radius. To assess the object area, an “area growing algorithm” connects the maximum with every adjacent pixel that has at least half the intensity of the local maximum. After defining the objects in the 53BP1 image, the software calculates object properties in the 53BP1 and the γH2AX images.

#### Derivation of an appropriate OEP

**Figure 2:**
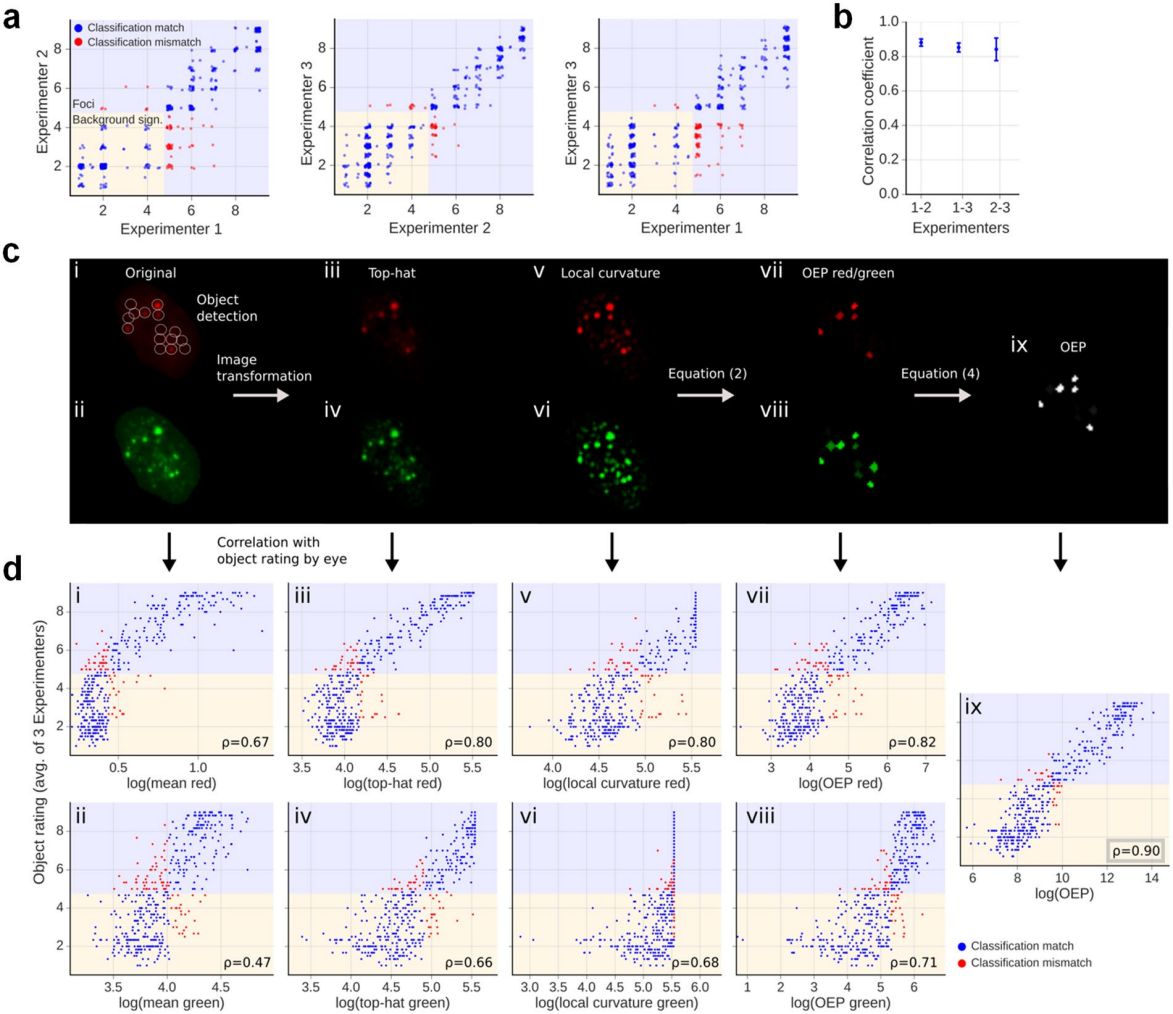
Derivation of an OEP that correlates best with manual object evaluation. Using single cell images, over 1,000 pre-defined objects from three independent experiments were manually rated on a scale from 1 to 9, where background signals were rated from 1 to 4 and foci from 5 to 9. (**a**) Representative comparison between object ratings from two experimenters. Blue dots indicate a matched classification while red dots indicate a mismatch regarding the classification of background signals vs. foci. Since the objects were not rated continuously but in steps of 0.5, a small random offset was added to visualize the frequency of objects in a certain category. (**b**) Rank correlation coefficients ρ between object ratings of two experimenters. The error bars represent the SD of three independent experiments. (**c**) Schematic depiction of the various steps used for the derivation of the OEP. The left two panels i and ii show the original IF images with the 53BP1 (red) and γH2AX (green) signals. The circles indicate the positions of the objects as defined in the 53BP1 image. Panels iii to vi show the IF images after the top-hat and the local curvature transformation. Panels vii to ix display the OEP for the defined objects according to equation (2) for the 53BP1 and γH2AX images or the combined OEP from the 53BP1 and γH2AX evaluation according to equation (4). (**d**) Scatter plots for the 53BP1 (red) and γH2AX (green) signals comparing the average manual object rating performed by the three experimenters for the representative experiment in panel a with results from the automated evaluation. The tested parameters were the average object intensity for panels i and ii, the average intensity of the three brightest pixels of each object in panels iii to vi or the analysis of the objects according to equations (2) or (4) for panels vii to ix. To allow a comparison between the automated results and the manual ratings, it was assumed that the evaluations using the tested parameters detect the same number of foci as the experimenters. The discrepancies in object classification are indicated by red dots. The logarithm for visualization of the automated results does not influence the rank correlation p and makes it easier to interpret the resulting histogram as the human perception usually follows a logarithmic scale (Weber-Fechner law)^48^.

We first compared various OEPs, calculated from object properties, with regard to their correlation with manual focus rating. Three experimenters created a manual benchmark by rating over 1,000 objects by eye on a scale from 1 to 9, where background signals were rated from 1 to 4 and foci from 5 to 9. The manual rating involved only a fraction of the background signals in order to have similar object numbers in each section of the rating scale. Figure 2a shows a comparison between the manual object ratings from different experimenters for one out of three performed experiments. About 15% of the objects classified as a focus by one experimenter are not classified as such by one of the other two experimenters. As a quantitative measure for the agreement between the object ratings of two different experimenters, we calculated the Spearman’s rank correlation coefficient ρ (Fig. 2b), which not only accounts for the classification into foci and background signals but also for the ordering within each of the classes. The correlation coefficients range from 0.78 to 0.91 with an average of 0.86, which served as a benchmark to assess the performance of a calculated OEP.

Figure 2c schematically depicts the relevant steps to obtain the OEP that correlates best with manual object rating. The correlations between the manual object ratings and the ratings obtained by the automated approach are presented in Fig. 2d. One simple OEP is the mean object intensity in the original image (Fig. 2c, panels i and ii). As shown in Fig. 2d, the correlation coefficients ρ are 0.67 for the 53BP1 (panel i) and 0.47 for the γH2AX signals (panel ii) and are substantially lower than those between different experimenters (compare to Fig. 2b). We therefore included two image transformations, a top-hat transformation and a transformation for the local curvature. The top-hat transformation was also used by previous methods for foci detection^31,32,35–37^ and enhances the intensity maxima of an image by subtracting the local background (Fig. 2c, panels iii and iv). Using the three brightest pixels within the objects in the top-hat transformed images as the OEP, the correlation coefficients between automated and manual ratings increase to 0.80 (53BP1; Fig. 2d, panel iii) and 0.66 (γH2AX; Fig. 2d, panel iv). The second image transformation, called “local curvature transformation”, applies a Laplacian of Gaussian (LoG) operator with a 5 × 5 kernel matrix (see Materials and Methods, User-defined parameters for AutoFoci). This transformation enhances regions with a rapid change of intensity, e.g. edges or high local curvatures, and is maximal for blob-like structures similar in size to the LoG kernel matrix (Fig. 2c, panels v and vi). Using the three brightest pixels within objects in the local curvature transformed images leads to correlation coefficients of 0.80 (53BP1; Fig. 2d, panel v) and 0.68 (γH2AX; Fig. 2d, panel vi). We then combined the results of both transformations and included a factor *C* that represents the object compactness (Fig. 2c, panels vii and viii), which leads to correlation coefficients of 0.82 (53BP1; Fig. 2d, panel vii) and 0.71 (γH2AX; Fig. 2d, panel viii). *C* is defined as the inverse of the objects’ “moment of inertia”, where the intensity distribution is used analogously to the mass distribution in the physical definition:

1 $$C=\frac{1}{{\sum }_{i}{r}_{i}^{2}{I}_{i}}$$

Here, *I*_*i*_ is the intensity of the i^th^ pixel within an object and *r*_*i*_ is its distance to the object centre. The complete OEP for one colour channel leads to the following formula:

2 $$OE{P}_{\text{red}/\text{green}}=\frac{{I}_{TH}}{{I}_{nucl}}\cdot {I}_{LC}\cdot C$$

Here, *I*_*TH*_ is the mean intensity of the three brightest pixels within an object in the top-hat transformed image, *I*_*nucl*_ the mean intensity of the nucleus and *I*_LC_ the mean intensity of the brightest three pixels within an object in the image after applying the local curvature transformation.

In the next step, we combined the two measurements obtained from the 53BP1 and γH2AX signals (Fig. 2c, panel ix). For this, we introduced a weighting factor *w* for the different staining qualities of the two markers, which is possibly considered unconsciously during manual focus evaluation:

3 $$w={I}_{{\rm{STDred}}}/{I}_{{\rm{STDgreen}}}$$

We calculated this factor for every cell individually and used it to increase the weight of the channel with the lower level of background signals. *I*_STDred_ and *I*_STDgreen_ denote the standard deviation of the pixel intensities within the nucleus for the 53BP1 and γH2AX signals, respectively. Typical values for *w* lie between 0.9 and 1.2 with most experiments showing a *w*-value above 1 that increases the weight of the 53BP1 signal. A combination of the OEPs for the 53BP1 and γH2AX signals by using this weighting factor results in the following formula:

4 $$OEP=OE{P}_{{\rm{red}}}^{w}\cdot OE{P}_{{\rm{green}}}^{\frac{1}{w}}$$

The resulting correlation value of 0.9 (Fig. 2d, panel ix) lies in the upper range found in the correlation between object evaluations by different experimenters. Therefore, an experimenter can rely more on the automated rating method to be similar to his own assessment than on one of his colleagues’. Since the automated rating demonstrates such a high correlation to the manual object rating, we used it for subsequent analyses.

#### Threshold estimation and manual validation

**Figure 3:**
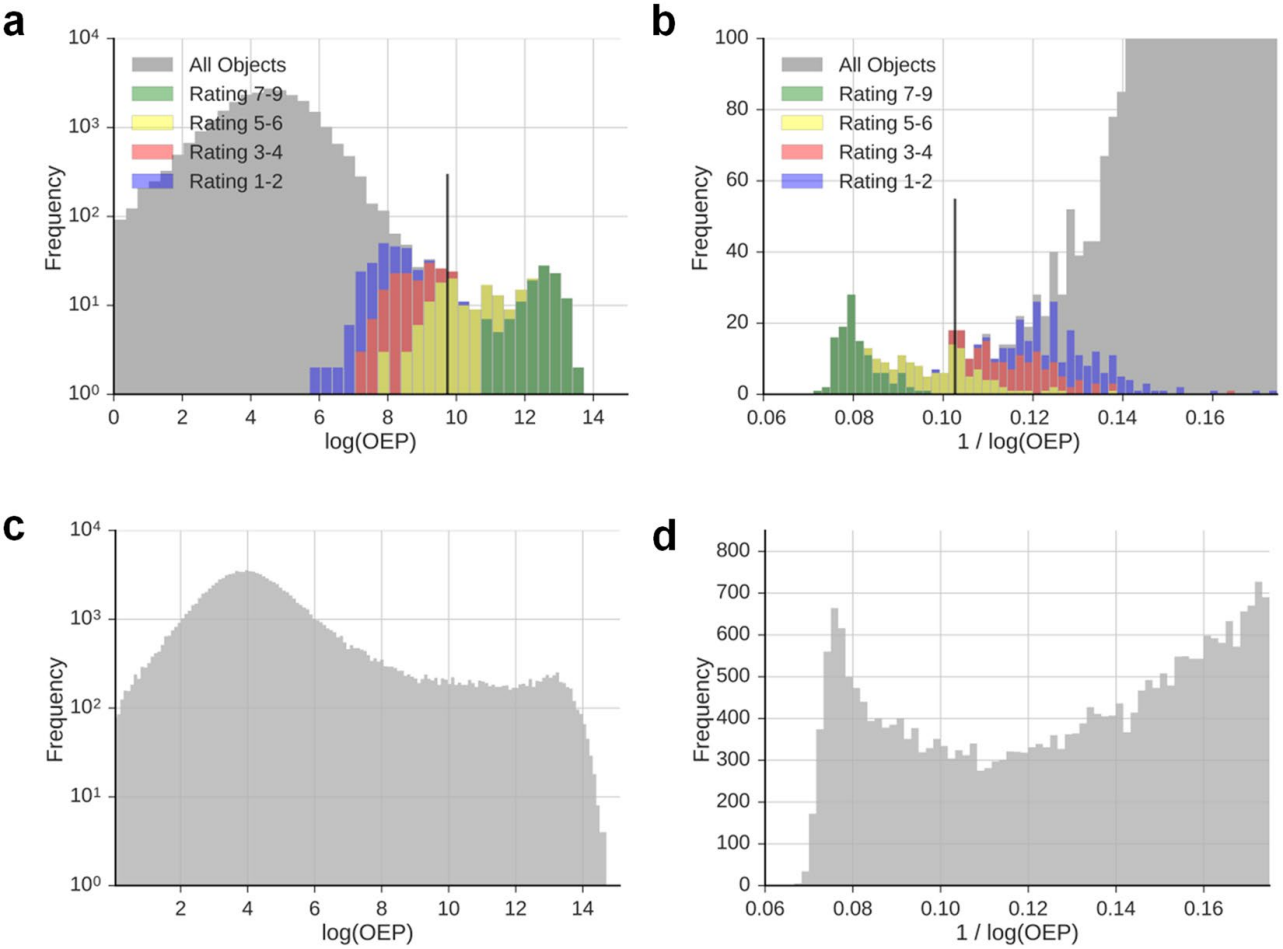
A bi-modal OEP histogram separating background signals and foci. Representative histograms showing the logarithmic OEP (**a**,**c**) or the inverse logarithmic OEP (**b**,**d**) for objects detected in unirradiated HOMSF1 cells (**a**,**b**) or HOMSF1 cells fixed at 24 h post 1 Gy (**c**,**d**). Coloured histograms in panels a and b show the results of the manual rating from Fig. 2a (average of the 3 experimenters). Since objects with OEP ratings near the manually defined foci threshold of 5 are located around the minimum in both representations, background signals and foci can be separated. The black lines in panels a and b indicate the mean of different algorithms determining the starting point for manual threshold adjustment.

To determine the threshold for distinguishing foci from background signals, we examined the distribution of OEP values in unirradiated cells with low foci numbers. Strikingly, the logarithmic OEP shows a histogram displaying a bi-modality (Fig. 3a), which is even more pronounced in the inverse representation of the logarithmic OEP (Fig. 3b). Bi-modal histograms are also observed for a dose of 1 Gy (Fig. 3c,d), suggesting that the identified OEP is also suitable for approaches investigating DSB repair after higher doses.

To test whether the minima of the OEP histograms separate foci from background signals, we compared the OEP histograms to the results of the manual object rating (depicted as coloured bars in Fig. 3a,b). Strikingly, the minima of the logarithmic and the inverse logarithmic OEP histograms nicely separate clearly visible foci (with object ratings >6) from clear background signals (object ratings <5) while objects with ratings 5 and 6 (i.e. those rated by experimenters to represent “borderline” foci) are located close to the minima. Therefore, we applied different algorithms to detect the transition point between foci and background signals and used the mean of these algorithms as a starting point for manual validation (see Supplementary Information). We performed this manual validation because the distributions of foci and background signals merge around the minimum and their distinction will not always coincide with the minimum position. The manual validation step starts by displaying the cells with the four closest objects around the starting threshold (see Fig. S2). The experimenter then rates objects as foci or background signal and the algorithm shifts the threshold dependent on this result and calculates the resulting number of foci per cell for the new threshold. Then, the algorithm displays the four objects around the new threshold and the experimenter repeats the evaluation step. To make the process as time efficient as possible, the algorithm initially shifts the threshold in large steps, which are gradually reduced with repeated iterations. The process ends when the standard deviation of the past six “foci per cell” values, calculated from the past six thresholds, is smaller than 5% of the average value. Finally, the program calculates the mean number of foci per cell from the last six thresholds.

The manual adjustment of the threshold for each sample also allows an evaluation of the staining quality of the analyzed data set. For this, the software displays the OEP histograms and calculates the Pearson coefficient p representing the mean correlation between the pixel intensities of both channels for all cell nuclei of the sample. Valid data sets show strong and specific intensity signals for both damage markers (Fig. S3a) resulting in a Pearson coefficient above 0.4 (empirical value derived from many experiments) and a pronounced minimum in the OEP histogram (Fig. S3b). In contrast, stainings with weak foci signals for one damage marker (Fig. S3c) don’t meet the Pearson criteria and don’t show a minimum in the OEP histogram (Fig. S3d). Stainings exhibiting many unspecific background signals can show a high Pearson coefficient (Fig. S3e) but also fail to show a minimum in the histogram (Fig. S3f). Collectively, these parameters identify experiments with staining or image acquisition problems that should be discarded.

### Inefficient DSB repair in human fibroblasts after low-dose exposure

**Figure 4:**
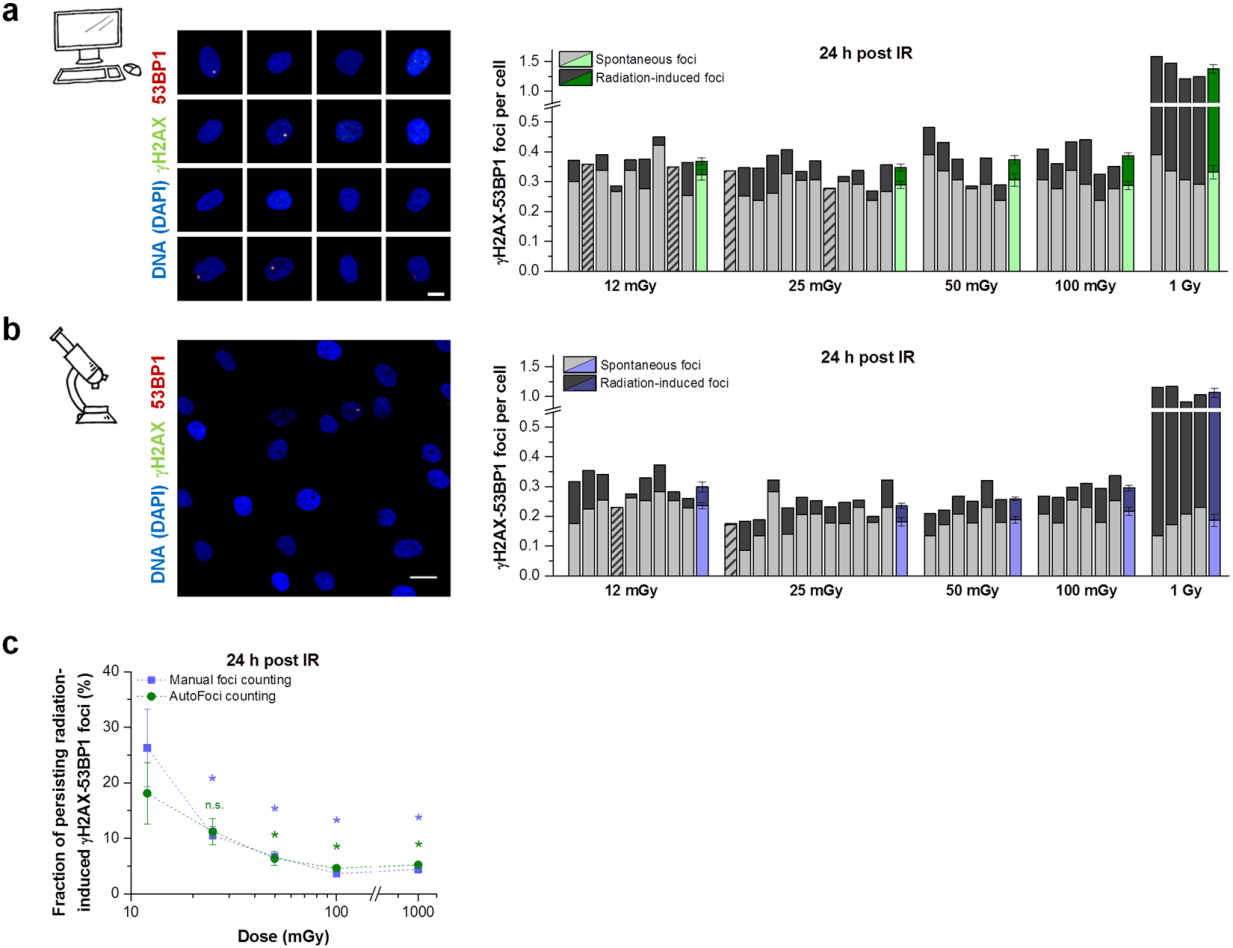
Inefficient DSB repair after irradiation with low X-ray doses. Non-dividing HOMSF1 cells were irradiated with various doses between 12 mGy and 1 Gy, fixed at 24 h after irradiation and stained for γH2AX, 53BP1 and DAPI. (**a**,**b**) Quantification of foci performed by AutoFoci (**a**) or manually by the experimenter (**b**). Left panels: For automated foci counting with AutoFoci, single cell images were used, while overview images containing many cells were used for manual foci counting. The scale bars represent 10 µm. Right panels: Each bar represents the mean foci number from 2 duplicate samples irradiated with the indicated dose (dark grey) plotted together with the corresponding mean foci number of unirradiated control samples (light grey). Striped bars indicate that the foci numbers of unirradiated and irradiated cells were similar (sparsely striped) or that irradiated cells showed slightly fewer foci numbers (<0.01 foci per cell) than the corresponding control (densely striped). For automated foci counting at least 5000 cells and for manual counting at least 1000 cells were analyzed per duplicate sample. Foci counting was performed in a blinded manner. The last column for each dose represents the mean value for unirradiated and irradiated cells from the shown 4–12 independent duplicates. Error bars represent the SE. (**c**) Evaluation of the DSB repair efficiency. The number of the radiation-induced persisting foci as shown in a and b (dark grey part of the columns) was divided by the number of foci induced at 15 min by the corresponding doses by applying an induction rate of 20 foci per cell for 1 Gy. Error bars show the SE from 4–12 duplicates and *indicates a p value < 0.05. Data sets for 12 mGy were tested against 25, 50, 100 and 1000 mGy. The detailed parameters are provided in Materials and Methods.

To test our new tool AutoFoci, we aimed to verify the previously observed inefficient DSB repair after low radiation doses^18,20^. We irradiated cells with doses between 12 mGy and 1 Gy, stained them 24 h later against γH2AX and 53BP1 and obtained ~5,000 single cell images per sample. For a direct comparison of the resulting AutoFoci data to a manual approach, we counted foci by eye in 500 cells per sample on microscopic overview images. Foci numbers obtained in irradiated samples by AutoFoci (Fig. 4a) and by manual counting (Fig. 4b) are plotted together with the corresponding numbers for unirradiated samples. In total, we analyzed foci in more than 600,000 cells using the AutoFoci approach and in about 50,000 cells manually. Although foci numbers vary between identical samples analyzed by the two approaches, the average number of radiation-induced persisting foci is very similar (compare the last coloured column for each dose in Fig. 4a,b). To assess the repair efficiency, we divided the number of radiation-induced persisting foci by the number of foci induced at 15 min by the corresponding doses. For this, we applied an induction rate of 20 foci per cell for a dose of 1 Gy (Fig. S1d), yielding 0.24 induced foci per cell for a dose of 12 mGy and proportionally higher values for the higher doses.

This analysis shows that only 4-5% of the initial foci persist for 24 h after 100 mGy and 1 Gy, suggesting efficient DSB repair at these higher doses (Fig. 4c). Strikingly, however, both quantification methods reveal that decreasing the dose below 100 mGy gradually decreases the repair efficiency (Fig. 4c). While about 10% of the initial foci persist for 24 h after 25 mGy, irradiation with 12 mGy leads to 18–25% persisting foci. These results fully confirm previous studies^18,20^ by applying our automated high-throughput foci quantification method to thousands of cells. Notably, the analysis requires the manual assessment of only a small subset of cells to evaluate foci numbers in the remaining thousands of cells in each sample. Thus, AutoFoci significantly speeds up the evaluation process while ensuring reliable results by including a manual control and adjustment step.

Although the numbers for persisting radiation-induced foci are very similar for the automated and the manual evaluation method, the foci numbers in unirradiated cells are significantly higher for the automated analysis (see Materials and Methods). A detailed analysis of the OEP histograms of unirradiated cells and of cells irradiated with 1 Gy reveals that the average OEP of persisting foci is considerably higher than that of spontaneous foci (Fig. S4). This indicates that spontaneous foci are on average smaller and less bright than the persisting radiation-induced foci. We speculate that the analysis of single cell images during AutoFoci leads to the classification of borderline objects as foci which are not classified as such at microscopic overview images used for manual counting.

## Discussion

Here, we present the software AutoFoci for automated high-throughput DSB scoring in human fibroblasts. We applied IF co-staining of γH2AX and 53BP1 foci to visualize DSBs. After automated image acquisition and processing, AutoFoci detects all objects, including foci as well as background signals, by local intensity maxima and additional object properties such as size and sharpness. Several experimenters rated the detected objects on a quality scale from 1 to 9 into foci (scores 5 to 9) and background signals (scores 1 to 4). This manual classification served as a benchmark to identify the combination of object properties defined as OEP in equation (4). The inverse logarithmic OEP shows a clear bi-modal object distribution with two maxima and a minimum in between that matches the transition between manually rated unspecific signals and foci. The software automatically identifies the minimum in the bi-modal OEP distribution which allows the separation of foci from background signals and hence enables automated DSB scoring.

Most other DSB scoring programs involve the manual setting of thresholds for various object properties to obtain average foci numbers per cell which match manual scoring but typically do not correlate the individual object properties, or a combination of them, with a manual classification (see Supplementary Information). Hence, the discrimination between foci and background signals based on a combination of object parameters which best correlates with a human classification represents a unique feature of the AutoFoci software. Importantly, the distributions obtained for the best combination of object properties as defined in the OEP exhibit a bi-modal pattern that allowed the automated scoring of foci. However, since the distributions for background signals and foci overlap around the minimum, we implemented a short manual evaluation to adjust the threshold. This brief human intervention has the additional advantage that it allows a manual assessment of image and staining quality, preventing the usage of data with insufficient quality. The time requirement for the intervention step is small since the method first estimates the threshold automatically and the subsequent manual validation involves only a small number of cells. Thus, the brief human adjustment step implemented in AutoFoci provides an ideal balance between speed and accuracy. Of note, the identified combination of object properties defined in the OEP can easily be implemented in most other DSB scoring programs.

To demonstrate the validity of our automated foci counting method AutoFoci for low-dose experiments, we analyzed the DSB repair efficiencies after irradiation of cultured fibroblasts with defined X-ray doses between 12 mGy and 1 Gy. Kept in confluence, unirradiated fibroblasts show foci numbers (representing spontaneous DSBs) which correspond to foci numbers induced by doses between 7–15 mGy. Thus, at the lowest dose used for the repair studies (12 mGy), irradiation approximately doubled the spontaneous foci level, allowing reliable assessment of repair efficiency. Previous studies succeeded in analyzing even lower doses enabled by a lower number of spontaneous foci^18,20^. The number of spontaneous foci detected might depend on cell type and cultivation, the optical resolution of the microscope and the type of evaluation (by eye at the microscope or at microscopic images). Importantly, by automatically quantifying foci in more than 600,000 cells, we confirmed that the efficiency of DSB repair decreases with decreasing radiation doses. At the lowest dose of 12 mGy, approximately 20% of the induced foci persist, which is consistent with manual quantifications of foci in the present and previous studies^18,20^.

Despite the demonstrated validity of our approach to count persisting foci after low radiation doses, program adjustments are required when earlier time points and/or higher doses need to be analyzed. At early times, foci are typically smaller and less intense compared with foci persisting at 24 h post IR. This will likely lead to a less distinct bi-modal OEP distribution which might cause difficulties to automatically identify the ideal separation point between foci and background signals. While the existing procedure for manual threshold adjustment can partially correct for this limitation, the algorithms for automated threshold estimation require adjustments. At higher doses, multiple cells will exhibit more than one focus which will not be adequately imaged by using a single plane. This will likely lead to an underestimation of foci numbers, a limitation which can be overcome by using maximum intensity projections (MIPs) for object detection. Thus, we have provided online instructions for changing the algorithms for automated threshold estimation as well as for using MIPs instead of single planes for object detection. Moreover, since the manual threshold adjustment procedure requires some level of experience by the experimenter, we have provided online the single cell images used for the manual object rating in Fig. 3 along with the classification of the pre-defined objects. Further, we have provided overview images that can be processed by Cellect for AutoFoci. This will allow users to compare their manual rating with the presented data set and will familiarize themselves with our software programs using appropriate images. It is also relevant to note that users can benchmark their foci rating by manually moving the threshold for foci detection in the OEP histograms into regions with clear foci or clear background signals.

The automated foci counting method provides a valuable tool for high-throughput image analysis in low-dose experiments. AutoFoci was applied in the present work to verify the previously observed inefficient DSB repair after low radiation doses but could prove useful for many other studies, particularly large-scale screening programs. For example, we are currently investigating the repair capacity of fibroblasts derived from patients who developed one or two independent malignancies before the age of eighteen. Such patients likely have a genetic predisposition to develop tumours which might arise due to differences in repairing low numbers of DSBs compared to healthy donors. Moreover, AutoFoci might be suitable for assessing the radiation sensitivity of cancer patients before receiving tumour therapy. Such studies are important to identify patients who likely will show severe side effects during radiotherapy because of an impaired DSB response^38–40^. Biodosimetry is another possible application of AutoFoci, where the dose an individual received is not known but can be approximated by measuring the level of induced DSBs. Such measurements help to optimize CT scanning protocols^38,41–43^ or to assess the dose levels during therapeutic approaches^44,45^. However, since most studies with human samples use lymphocytes, the described method of image acquisition and processing requires adjustments. Since AutoFoci provides an ideal balance between speed and accuracy, it might improve the feasibility and validity of such large-scale studies.

## Materials and Methods

### Cultivation of human fibroblasts

HOMSF1 human fibroblasts (a kind gift from the Human Genetics Department at the University of Saarland, Germany) were cultured in DMEM (low glucose, supplemented with 15% FCS, 1% NEAA, 100 units/ml penicillin, 0.1 mg/ml streptomycin and 0.25 µg/ml ampothericin) at 37 °C and 5% CO_2_. Cells in passages 11 to 16 were used for the experiments in which about 10,000 cells per cm^2^ were seeded either in 24-well plates with a glass bottom or on glass cover-slips. Cells were cultured for two weeks to obtain a non-dividing confluent cell layer. During this incubation time, the cell culture medium was exchanged once.

### Cell cycle analysis

To control for the non-dividing status of the confluent cell layer, cells were evaluated by eye and routinely tested by cell cycle analysis. For this, resting and exponentially growing cells were incubated for 3 h with 5 µM of the nucleotide analogue EdU. Cells were fixed using 3% para-formaldehyde and permeabilized with 0.2% Triton in PBS for 10 min at RT. EdU incorporated during the S phase of the cell cycle was stained via a click-it reaction according to the manufacturer’s instructions (PANATecs). DNA was stained with DAPI (0.2 µg/ml) for 5 min. Cells were washed again with ultrapure water, mounted with VECTASHIELD mounting medium (Vector Laboratories) and sealed with nail polish. The population was automatically scanned at a widefield microscope (Axiovert 200 M with a 20x Plan-Apochromat objective, Zeiss) and the EdU signal intensity was plotted vs. the DAPI signal intensity by using the program MetaCyte (MetaSystems). Non-dividing G0/G1 cells have a low EdU and a low DAPI signal (Fig. S1a,b).

### Irradiation of HOMSF1 cells

Irradiation of HOMSF1 cells was performed with an X-ray machine (Titan Isovolt 160; General Electric) at 90 kV, 3 mA and a dose rate of 30 mGy per min (for doses <20 mGy), 6 mA and a dose rate of 60 mGy per min (for doses >20 and <100 mGy) or 19 mA and a dose rate of 550 mGy per min (for doses ≥100 mGy). The dose rate was determined and controlled with a dosimeter (DIADOS T60004; PTW Freiburg GmbH). Since cells were irradiated on a glass surface, a dose correction factor of 3 was applied, which was determined experimentally according to a previous study^46^.

### γH2AX/53BP1 immunofluorescence staining, image acquisition and processing

At defined times post irradiation, cells were washed once with PBS and fixed with 3% paraformaldehyde for 15 min. Cells were washed again with PBS and permeabilized with 0.2% Triton in PBS for 10 min at RT. After three washing steps with PBS, cells were incubated with blocking-solution (1% FCS and 3% BSA in PBS) for 30 min at RT. Cells were incubated with anti-53BP1 antibody (mouse; Upstate 24568) at 1:2000 and anti-γH2AX antibody (rabbit; Abcam GR1377) at 1:1000 in PBS containing 1% FCS at 4 °C over night. Cells were washed three times with PBS-T (0.1% Tween20 in PBS) and incubated with goat-anti-rabbit and goat-anti-mouse antibody (Alexa Fluor 488 or 594; Invitrogen) at 1:500 or 1:1000 in PBS containing 1% FCS for 1 h at RT in the dark. After another three washing steps with PBS, DNA was stained with DAPI (0.2 µg/ml) for 5 min. Cells were washed again with ultrapure water, mounted with VECTASHIELD mounting medium (Vector Laboratories) and sealed with nail polish.

For image acquisition, a scan raster of 5 × 5 or 10 × 10 fields each with a size of 1360 × 1024 pixels (439.28 × 330.75 µm^2^) was applied. For each field, 1 image for DAPI and z-stacks of 5 images for each damage marker with a z-distance of 1.2 µm were obtained at a widefield microscope (Observer D1, Zeiss) with a 20x Plan-Apochromat objective (Zeiss). Images were captured automatically utilizing the autofocus feature of the µManager software (Vale lab, UCSF) operating with the JAF(H&P) algorithm and optimized autofocus properties for the used cell line.

For automated foci counting via AutoFoci, images were processed with Cellect to generate single cell images. For this, the position information of the cells was determined by their DAPI signal, which was then used to crop each cell to obtain single cell images. To select the best of the five planes, transformed images were created by applying the Sobel operator that enhances the edges within the image which correspond to foci and the nuclear membrane. The best plane is then defined as the plane with the highest total intensity within the nucleus in the transformed image compared to the total intensity in the original image. These steps were performed for each cell individually. Finally, a histogram displaying the DAPI intensity vs. the intensity of the γH2AX signal in the nucleus was used to select the G1 population and to exclude S and G2 cells as well as any potentially dying cells by their abnormal DAPI and γH2AX signals. For further details see the guideline for image processing using Cellect provided at https://github.com/nleng/AutoFoci.

### Manual foci counting and threshold adjustment for automated foci counting

Manual foci counting was typically performed in a blinded manner using overview images. For repair studies at 24 h post irradiation, single overview images (the plane identified by the autofocus function during image acquisition) were used to quantify foci, whereas z-stacks were used to count foci at 15 min post irradiation because of the smaller foci size directly after irradiation.

The evaluation of the critical objects during threshold adjustment for automated foci counting was typically performed in a blinded manner. For this, images of several samples were opened at once and the experimenter was not aware of which sample was selected for threshold adjustment. For further details, see the guideline to operate AutoFoci provided at https://github.com/nleng/AutoFoci.

### User-defined parameters for AutoFoci

The software AutoFoci requires only a small number of user-defined input parameters. These parameters depend on the resolution of the cell images, which was 120 × 120 pixels in this study. One of the parameters is the so-called local maximum radius which was used for object separation. A radius of 3 pixels was used in the data presented here. It only had a small influence on the number of detected foci as only very few foci were closer than 3 pixels to each other, especially in the performed repair studies after low doses. Additionally, we used a value of 3 pixels for the minimum area of an object and a factor of 1.1 for the minimum intensity of each pixel (within an object) above the mean intensity of the cell nucleus (selected for 8-bit images with grey values of 0–255). Because both were set to very small values, they did not influence the number of foci and were only used to reduce the number of background signals. A lower number of background signals reduces the CPU usage and also the required disk space. As a second parameter, the diameter of the so-called structuring element applied for the top-hat transformation should be larger than the diameter of a small focus and was set to 10 pixels.

For the image transformation “local curvature”, we used the following 5 × 5 kernel matrix to approximate a Laplacian of Gaussian (LoG) operator:

$$(\begin{array}{ccccc}-2 & -4 & -4 & -4 & -2\\ -4 & 0 & 10 & 0 & -4\\ -4 & 10 & 32 & 10 & -4\\ -4 & 0 & 10 & 0 & -4\\ -2 & -4 & -4 & -4 & -2\end{array})$$

The convolution matrix acts as a Gaussian smoothing filter followed by the Laplace operator, which approximates a second derivative measurement on the image^47^. The Gaussian smoothing was applied before the Laplacian operator because the latter is sensitive to noise.

### Statistical Analysis

Statistical analyses were carried out by applying a one-sided t-test with a significance level of 0.05. Data sets were tested for their normal distribution. Variances of data sets for t-testing were first compared via F-test. If the variances of the data sets differed significantly, a Welch-Correction was included in the t-test. All details such as sample size, 95% confidence interval (CI), degree of freedom (DF) and p-value are noted below. We performed all statistical analyses with OriginPro 9.0 G (OriginLabs).

Average spontaneous foci numbers per cell quantified by the manual vs. the automated approach (Fig. 4a,b): p < 0.001, DF40. Descriptive statistics: AutoFoci counting: N = 26; 0.30 ± 0.01 (SE), 0.28–0.33 (CI). Manual counting: N = 26, 0.21 ± 0.01 (SE), 0.18–0.23 (CI).

Fraction of persisting radiation-induced foci after 12 mGy vs. higher radiation doses (Fig. 4c): AutoFoci counting: 12 vs. 25, 50, 100, 1000 mGy: p = 0.138, DF = 11; p = 0.0346, DF = 8.18; p = 0.020, DF = 8.13; p = 0.024, DF = 8.06. Manual counting: 12 vs. 25, 50, 100, 1000 mGy: p = 0.010, DF = 19; p = 0.020, DF = 13; p = 0.006, DF = 8.07; p = 0.007, DF = 8.05. Descriptive statistics: AutoFoci counting: 12 mGy: N = 9, 18.12 ± 5.5 (SE), 5.36–30.87 (CI); 25 mGy: N = 12, 11,22 ± 2.40 (SE), 5.95–16.49 (CI); 50 mGy: N = 6, 6.39 ± 1.23 (SE), 3.14–9.64 (CI); 100 mGy: N = 6, 4.62 ± 0.50 (SE), 3.33–5.91 (CI); 1000 mGy: N = 4, 5.23 ± 0.35 (SE) or 4.13–6.33 (CI). Manual counting: 12 mGy: N = 9, 26.29 ± 6.9 (SE), 10.28–42.30 (CI); 25 mGy: N = 12, 10,50 ± 1.67 (SE), 6.82–14.18 (CI); 50 mGy: N = 6, 6.71 ± 0.57 (SE), 5.25–8.16 (CI); 100 mGy: N = 6, 3.6 ± 0.45 (SE), 2.49–4.81 (CI); 1000 mGy: N = 4, 4.41 ± 0.38 (SE), 3.20–5.62 (CI).

## Electronic supplementary material

Supplementary information

## Data Availability

The ImageJ version including the Cellect tools used for image processing and the software AutoFoci to automatically count foci in single cell images are freely available at https://github.com/nleng/AutoFoci. Furthermore, short guidelines and test images for both programs are provided on the website for trial.

## References

[1] Federal Office of Radiation Protection. Wie hoch ist die natürliche Strahlenbelastung in Deutschland? *Available from*, http://www.bfs.de/DE/themen/ion/umwelt/natuerliche-strahlenbelastung/natuerliche-strahlenbelastung.html (2016).

[2] Fazel R (2009). Exposure to low-dose ionizing radiation from medical imaging procedures. N Engl J Med.

[3] Smith-Bindman R (2009). Radiation dose associated with common computed tomography examinations and the associated lifetime attributable risk of cancer. Arch Intern Med.

[4] Davis AJ, Chen DJ (2013). DNA double strand break repair via non-homologous end-joining. Transl Cancer Res.

[5] Jeggo PA, Löbrich M (2015). How cancer cells hijack DNA double-strand break repair pathways to gain genomic instability. Biochem J.

[6] Mladenov E, Magin S, Soni A, Iliakis G (2016). DNA double-strand-break repair in higher eukaryotes and its role in genomic instability and cancer: Cell cycle and proliferation-dependent regulation. Semin Cancer Biol.

[7] Meulepas JM, Hauptmann M, Lubin JH, Shuryak I, Brenner DJ (2018). Is there unmeasured indication bias in radiation-related cancer risk estimates from studies of computed tomography?. Radiat Res.

[8] Mullenders L, Atkinson M, Paretzke H, Sabatier L, Bouffler S (2009). Assessing cancer risks of low-dose radiation. Nat Rev Cancer.

[9] Brenner DJ (2010). Should we be concerned about the rapid increase in CT usage?. Rev Environ Health.

[10] Brenner DJ (2014). What we know and what we don’t know about cancer risks associated with radiation doses from radiological imaging. Br J Radiol.

[11] Doss M (2014). Radiation doses from radiological imaging do not increase the risk of cancer. Br J Radiol.

[12] Biehs R (2017). DNA double-strand break resection occurs during non-homologous end joining in G1 but is distinct from resection during homologous recombination. Mol Cell.

[13] Löbrich M, Jeggo PA (2017). A process of resection-dependent nonhomologous end joining involving the goddess Artemis. Trends Biochem Sci.

[14] Meers C, Keskin H, Storici F (2016). DNA repair by RNA: Templated, or not templated, that is the question. DNA Repair.

[15] Jackson SP, Helleday T (2016). DNA REPAIR. Drugging DNA repair. Science.

[16] Asaithamby A, Chen DJ (2009). Cellular responses to DNA double-strand breaks after low-dose γ-irradiation. Nucleic Acids Res.

[17] Barazzuol L, Jeggo PA (2016). *In vivo* sensitivity of the embryonic and adult neural stem cell compartments to low-dose radiation. J Radiat Res.

[18] Grudzenski S, Raths A, Conrad S, Rube CE, Löbrich M (2010). Inducible response required for repair of low-dose radiation damage in human fibroblasts. Proc Natl Acad Sci USA.

[19] Mirsch J (2015). Direct measurement of the 3-dimensional DNA lesion distribution induced by energetic charged particles in a mouse model tissue. Proc Natl Acad Sci USA.

[20] Rothkamm K, Löbrich M (2003). Evidence for a lack of DNA double-strand break repair in human cells exposed to very low x-ray doses. Proc Natl Acad Sci USA.

[21] Schanz S (2012). Accumulation of DNA damage in complex normal tissues after protracted low-dose radiation. DNA Repair.

[22] Kuefner MA (2012). Effect of antioxidants on X-ray-induced gamma-H2AX foci in human blood lymphocytes: Preliminary observations. Radiology.

[23] Osipov AN (2015). Low doses of X-rays induce prolonged and ATM-independent persistence of gammaH2AX foci in human gingival mesenchymal stem cells. Oncotarget.

[24] Rogakou EP, Pilch DR, Orr AH, Ivanova VS, Bonner WM (1998). DNA double-stranded breaks induce histone H2AX phosphorylation on serine 139. J Biol Chem.

[25] Löbrich M (2010). GammaH2AX foci analysis for monitoring DNA double-strand break repair: Strengths, limitations and optimization. Cell Cycle.

[26] Rogakou EP, Boon C, Redon C, Bonner WM (1999). Megabase chromatin domains involved in DNA double-strand breaks *in vivo*. J Cell Biol.

[27] Sedelnikova OA, Rogakou EP, Panyutin IG, Bonner WM (2002). Quantitative detection of (125)IdU-induced DNA double-strand breaks with gamma-H2AX antibody. Radiat Res.

[28] Durdik M (2015). Imaging flow cytometry as a sensitive tool to detect low-dose-induced DNA damage by analyzing 53BP1 and gammaH2AX foci in human lymphocytes. Cytometry A.

[29] Jucha A (2010). FociCounter: A freely available PC programme for quantitative and qualitative analysis of gamma-H2AX foci. Mutat Res.

[30] Carpenter AE (2006). CellProfiler: Image analysis software for identifying and quantifying cell phenotypes. Genome Biol.

[31] Ivashkevich AN (2011). γH2AX foci as a measure of DNA damage: A computational approach to automatic analysis. Mutat Res.

[32] Qvarnström OF, Simonsson M, Johansson K-A, Nyman J, Turesson I (2004). DNA double strand break quantification in skin biopsies. Radiother Oncol.

[33] Runge R (2012). Fully automated interpretation of ionizing radiation-induced γH2AX foci by the novel pattern recognition system AKLIDES. Int J Radiat Biol.

[34] Rothkamm K (2013). Manual versus automated gamma-H2AX foci analysis across five European laboratories: Can this assay be used for rapid biodosimetry in a large scale radiation accident?. Mutat Res.

[35] Roch-Lefèvre S (2010). Quantification of gH2AX foci in human lymphocytes: A method for biological dosimetry after ionizing radiation exposure. Radiat Res.

[36] Valente M, Voisin P, Laloi P, Roy L, Roch-Lefèvre S (2011). Automated gammaH2AX focus scoring method for human lymphocytes after ionizing radiation exposure. Radiation Measurements.

[37] Böcker W, Iliakis G (2006). Computational methods for analysis of foci: Validation for radiation-induced gamma-H2AX foci in human cells. Radiat Res.

[38] Löbrich M (2005). *In vivo* formation and repair of DNA double-strand breaks after computed tomography examinations. Proc Natl Acad Sci USA.

[39] Löbrich M, Kiefer J (2006). Assessing the likelihood of severe side effects in radiotherapy. Int J Cancer.

[40] Schuler N (2014). DNA-damage foci to detect and characterize DNA repair alterations in children treated for pediatric malignancies. PloS One.

[41] Grudzenski S, Kuefner MA, Heckmann MB, Uder M, Löbrich M (2009). Contrast medium-enhanced radiation damage caused by CT examinations. Radiology.

[42] Kuefner MA (2010). Effect of CT scan protocols on x-ray-induced DNA double-strand breaks in blood lymphocytes of patients undergoing coronary CT angiography. Eur Radiol.

[43] Rothkamm K, Balroop S, Shekhdar J, Fernie P, Goh V (2007). Leukocyte DNA damage after multi–detector row CT: A quantitative biomarker of low-level radiation exposure. Radiology.

[44] Eberlein U, Peper M, Fernandez M, Lassmann M, Scherthan H (2015). Calibration of the gamma-H2AX DNA double strand break focus assay for internal radiation exposure of blood lymphocytes. PloS One.

[45] Zahnreich S, Ebersberger A, Kaina B, Schmidberger H (2015). Biodosimetry based on gamma-H2AX quantification and cytogenetics after partial- and total-body irradiation during fractionated radiotherapy. Radiat Res.

[46] Kegel P, Riballo E, Kuhne M, Jeggo PA, Löbrich M (2007). X-irradiation of cells on glass slides has a dose doubling impact. DNA Repair.

[47] Basu M (2002). Gaussian-based edge-detection methods-a survey. IEEE Transactions on Systems, Man, and Cybernetics, Part C.

[48] Hecht S (1924). The visual discrimination of intensity and the Weber-Fechner law. J Gen Physiol.

