# Supplementary material for: AutoFoci, an automated high-throughput foci detection approach for analyzing low-dose DNA double-strand break repair
Source: Sci Rep. 2018 Nov 23;8:17282. doi: 10.1038/s41598-018-35660-5 (PMC6251879; doi:10.1038/s41598-018-35660-5)
Supplement: Supplementary file 1 — Supplementary information [file 41598_2018_35660_MOESM1_ESM.pdf]

Supplementary Information for:

AutoFoci, an automated high-throughput foci detection approach for analyzing low-dose DNA double-strand break repair

Nicor Lengert[#,*], Johanna Mirsch[#], Ratna N. Weimer, Eik Schumann, Peter Haub, Barbara Drossel and Markus Löbrich*

# shared first author

*Correspondence to Nicor Lengert (nicor@fkp.tu-darmstadt.de) or

Markus Löbrich (lobrich@bio.tu-darmstadt.de)

Supplementary figures and figure legends

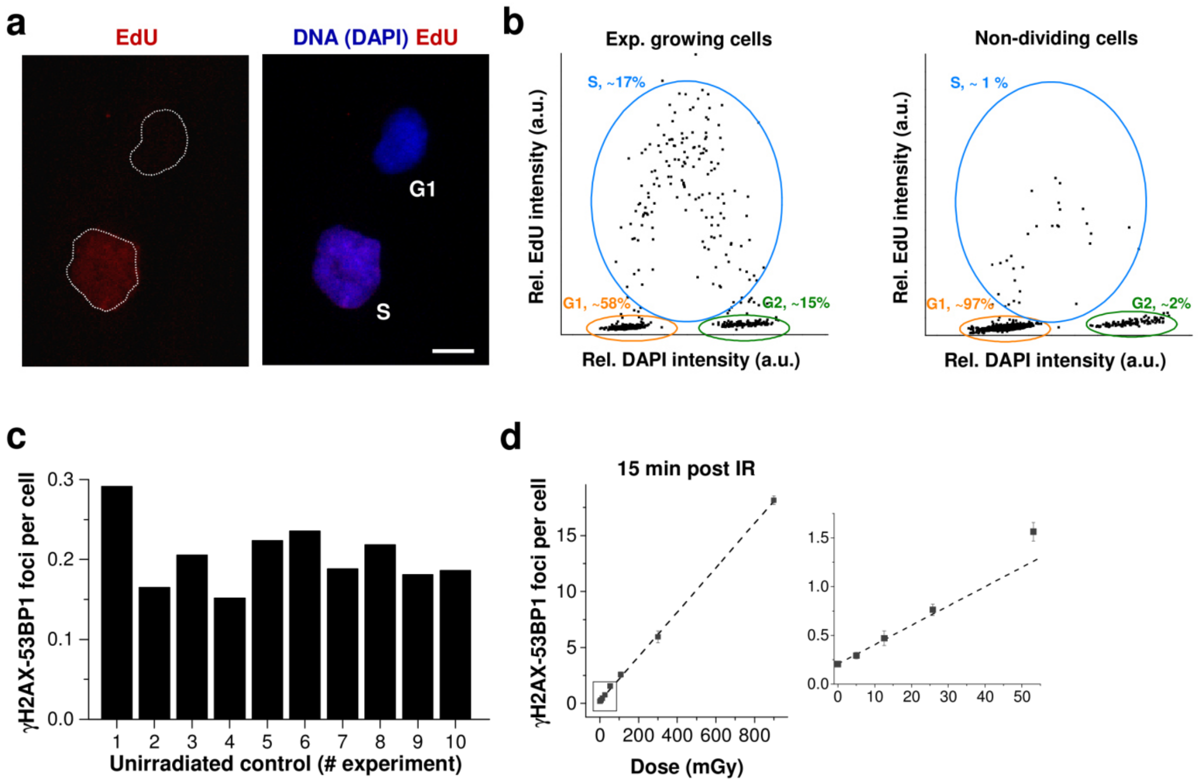

Figure S1: Characterization of HOMSF1 cells. (**a, b**) For cell cycle analysis, both exponentially growing and non-dividing HOMSF1 cells were incubated for 3 h with 5 µM EdU, fixed and stained for EdU and DAPI. (a) EdU staining of exponentially growing HOMSF1 cells. Dotted lines indicate the shape of the nuclei. The scale bar represents 10 µm. (b) Cell cycle distribution of exponentially growing (left panel) and non-dividing (right panel) HOMSF1 cells. The intensity of the EdU and DAPI signal was measured at the microscope for each cell. For the displayed distribution, around 1000 (left panel) or 4000 cells (right panel) were analyzed. (**c, d**) Quantification of spontaneous and radiation-induced foci in confluent HOMSF1 cells. HOMSF1 cells were left unirradiated (c) or were irradiated with various doses (d), fixed at 15 min post IR and stained for γH2AX, 53BP1 and DAPI. For each dose, n≥3 independent experiments were performed in duplicate samples. For each sample, foci were quantified manually in 200-500 cells (if the average number of foci per cell was below 1), in 100 cells (if the average number of foci per cell was below 10) or in 40 cells (if the average number of foci per cell was larger than 10) in a blinded manner at microscopic overview images. Data show the mean foci number per cell of 10 duplicate samples (c) or the mean of 3-7 duplicates (d). Error bars in panel d represent the SE. The regression curve f(x)=0.020x+0.21 (f: foci per cell; x: dose in mGy; R^2=0.999) was obtained by linear fitting.

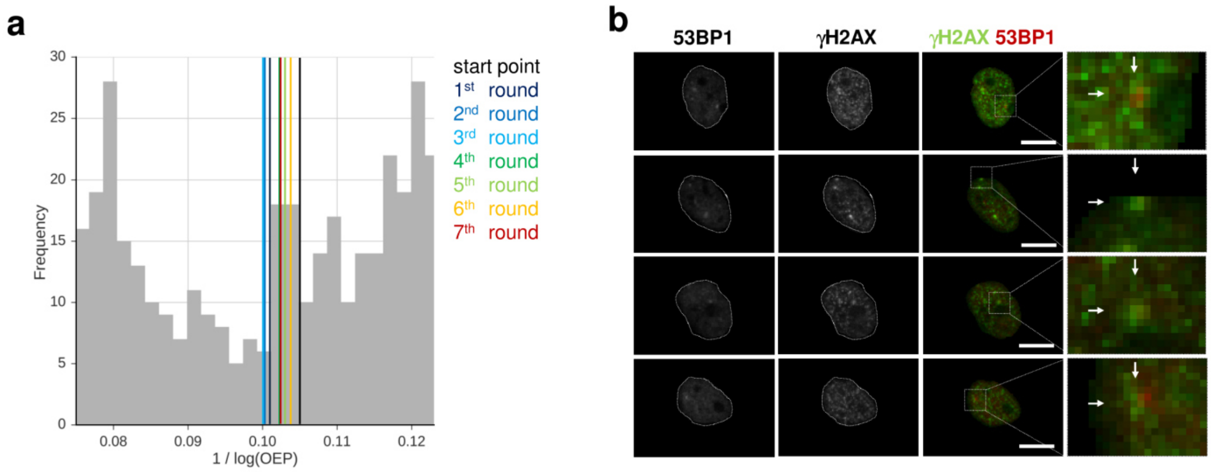

Figure S2: Steps for threshold validation to discriminate background signals from foci. (**a**) For threshold estimation, the mean value of nine different algorithms is used to automatically detect the transition point between foci and background signals in OEP histograms. This mean value, indicated as black line in the histogram, is used as a starting point for manual threshold adjustment. (**b**) For this, AutoFoci displays four cells each showing one critical object at the actual threshold in the red (53BP1), the green (γH2AX) and the merged images. White arrows mark the critical objects in a higher magnification image. The experimenter evaluates the four objects manually and enters the number of identified foci in the program. An algorithm then shifts the threshold depending on the manual input (indicated as dark blue line for the first evaluation round) and displays again four critical objects at the new threshold for the second evaluation round. The threshold is initially shifted in large steps, which are gradually reduced with repeated validation steps. The process stops when at least 24 cells (6 rounds) are evaluated and the standard deviation of the last six "foci per cell" values, calculated from the mean of the last six thresholds, is smaller than 5% of the average value. The scale bars represent 10 µm. Dotted lines indicate the shape of the nuclei.

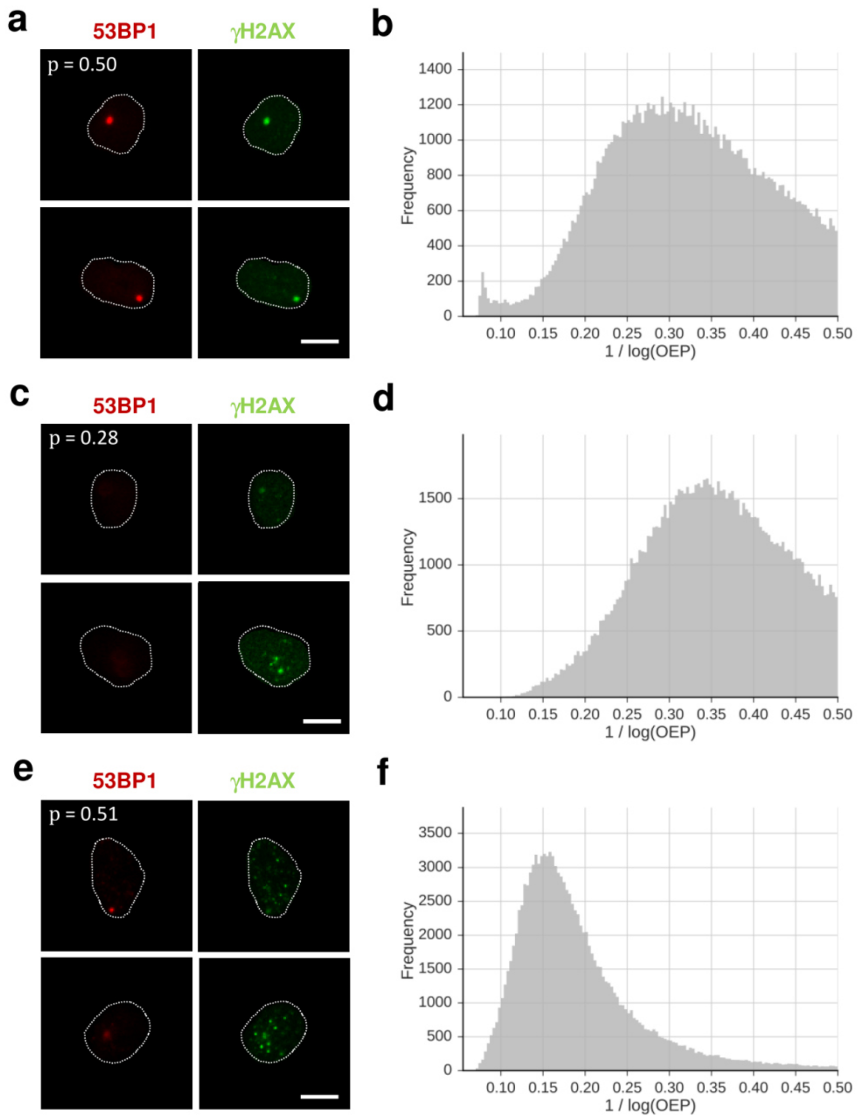

Figure S3: Use of OEP histograms for quality control. (**a, c, e**) Representative IF images of γH2AX and 53BP1 stainings in unirradiated non-dividing HOMSF1 cells with a good staining quality (a), a weak red signal (c) or many bright background signals in the green channel (e). Dotted lines indicate the shape of the nuclei. The scale bars represent 10 µm and the Pearson coefficients p show the correlation between the pixel intensities in both channels for all nuclei of the sample. (**b, d, f**) Inverse logarithmic OEP distributions from the samples in the left panels. The OEP was measured for each object as described by equation (4) in the main text.

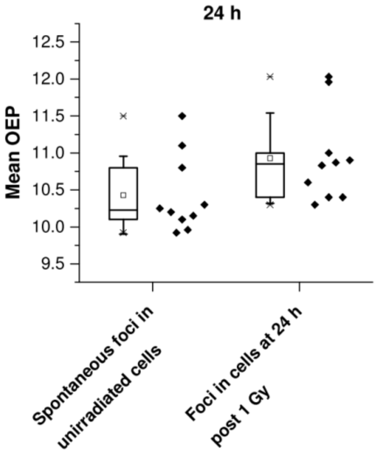

Figure S4: Lower OEPs for spontaneous than for radiation-induced foci. OEP values were obtained in unirradiated cells with spontaneous foci and in cells at 24 h after 1 Gy where the persisting radiation-induced foci outnumber the spontaneous foci. The whiskers of the box plots (25-75%) represent the SD between the measured OEPs of 10 samples each. The ranges between the "x" below and the "x" above the boxes encompass 98% of the data.

Algorithms for automated threshold estimation

Before manual validation of the threshold, which is later used for classification into foci and background signals, the threshold is first estimated by the mean value of nine different automated threshold methods. All algorithms are applied to the logarithmic OEP histograms unless stated otherwise.

Pixel correlation method

Because the co-localisation of both markers (γH2AX and 53BP1) is used as criterion for a DSB, we assumed that the correlation between the pixel values of both markers (Pearson coefficient) is higher in nuclei containing foci compared to nuclei without foci. Therefore, this method calculates the average Pearson correlation coefficients for both groups of cells for a range of potential thresholds. The best threshold is then estimated at the position where the difference between both coefficients has its maximum.

Poisson distribution method

This method assumes that the distribution of foci per cell above the correct threshold is closer to an ideal Poisson distribution than the distribution above a lower threshold. The deviation from an ideal Poisson distribution is calculated as the Kulback-Leibler (KL) divergence for a range of potential thresholds. The algorithm then estimates a threshold by smoothing the resulting diagram of KL divergences and applying another threshold method (maximum entropy) to it.

STD method: Minimum estimation by maximum standard deviation (STD)

The minimum between the two peaks of the bi-modal histogram is estimated by calculating the standard deviation of a defined number of consecutive objects. The value with the highest standard deviation, i.e. where the objects are farthest apart, is assumed to be the minimum. This method is applied to the histogram of the inverse representation of the logarithmic OEP where the bi-modal characteristic is more pronounced.

Range method: Minimum estimation by maximum object number in a given range

Very similar to the STD method, this method tries to find the minimum between two peaks in a given histogram. It counts the number of objects in a defined range around a threshold and estimates the minimum of the histogram at the position where the number of counted objects has its minimum. It is also applied to the inverse representation of the logarithmic OEP.

Maximum entropy method

This methods implements the Kapur-Sahoo-Wong thresholding method[1]. It calculates the Shannon entropy after classification of the objects into two groups by using a preliminary

threshold. The final threshold is assumed to be at the position of maximum entropy. The ImageJ implementation was used for this method.

Rényi entropy method

This method is similar to the maximum entropy method, but here the definition of entropy is modified slightly (Rényi entropy). Again, the threshold is assumed to be at the position where the entropy is at its maximum. The ImageJ implementation was used for this method.

Yen method

Here, Yen's thresholding method is used[2]. It calculates a correlation criterion and assumes the threshold to be at the maximum of this criterion. The ImageJ implementation was used for this method.

Triangle method

The Triangle algorithm is a geometric method[3], which can be used for distributions with one large peak and a long tail. It is therefore applied to the non-logarithmic OEP. It calculates a straight line between the peak of the histogram and the end of the tail. The threshold is then estimated at the position where the vertical distance between the calculated line and the histogram is maximal. The ImageJ implementation was used for this method.

Intermode method

This method assumes a bi-modal histogram with two peaks[4]. Iteratively, smoothing is applied to the histogram until only two local maxima exist. The threshold is assumed to be the middle point between both local maxima. The ImageJ implementation was used for this method.

Discussion of AutoFoci features in the context of existing programs

Although automated foci counting methods exist, we had especially strict requirements for our low-dose experiments. The low-dose specific requirements include high-speed analysis and accurate differentiation between foci and background signals. Here, we provide a discussion of AutoFoci in the context of other existing computer programs for foci counting.

The CellProfiler project, a versatile platform for image analysis[5], allows to create a pipeline with a foci detection algorithm very similar to the one presented here. However, the CellProfiler needs a few hours to analyze 5,000 cells because it stores and analyzes more data than necessary, while our software AutoFoci only needs 30 to 60 s (plus the time to validate the threshold by inspecting about 24-40 cells). Moreover, CellProfiler uses a simple threshold method, termed Otsu method, which is usually not sufficient to discriminate between background signals and foci. Thus, the threshold has to be corrected by a certain factor[6] for each sample, which makes a comparison between different samples difficult.

The definition of a focus by the commercial AKLIDES® system, which provides a fully automated detection of γH2AX foci, is very similar to our definition of a local maximum: A pixel brighter than its neighbours with a minimal relative brightness and a minimal distance to other local maxima[7,8]. The intensity and size thresholds of foci are specific for each cell line but from the available publications it is not clear how they are obtained and whether it requires human intervention.

Similar to the method presented here, Qvarnström *et al.* and Böcker *et al.* use a top-hat transformation of the γH2AX signal[9,10]. A simple global intensity threshold is subsequently used for foci classification, which would result in a correlation to manual foci evaluation comparable to the reported value of the green channel after top-hat transformation (ρ=0.66; Fig. 2c) when applied to data of similar staining quality as presented here. Like Qvarnström *et al.,* the method presented by Ivashkevich *et al.* and its further development by Jakl *et al.* uses a top-hat transformation of the γH2AX signal, but then applies an additional image filter (H-dome transformation) before setting the intensity threshold[11,12]. This threshold is determined by a very interesting approach, maximizing the difference between the number of objects counted as foci in the images of irradiated and non-irradiated cells. An essential requirement for this method is that both samples are treated completely identically, which is often hard to achieve. Another requirement for this approach is that the foci in the irradiated samples occupy a considerable fraction of the total nuclear area such that non-irradiated cells exhibit more background signals than irradiated cells. Otherwise, the difference would not decrease at small threshold values. Additionally, maximizing the difference when the difference itself is the desired result may lead to a systematic error. Nevertheless, we applied the method to our data from primary human fibroblasts but found that the maximum was generally at much too large values because of the heterogeneity of the distribution of background signals even for cell samples that were treated identically.

Herbert *et al.* solve the threshold question by using a machine learning algorithm to determine suitable foci parameters[13]. Similar to the threshold validation approach in our method, the learning process requires human intervention for every experiment. However, the time requirement of our method is considerably lower since it first estimates the threshold automatically and the manual validation step involves only a small number of critical objects.

References

1 Kapur, J. N., Sahoo, P. K. & Wong, A. K. C. A new method for gray-level picture thresholding using the entropy of the histogram. *Computer vision, graphics, and image processing* 29, 273-285 0.1016/0734-189X(85)90125-2 (1985).

2 Yen, J.-C., Chang, F.-J. & Chang, S. A new criterion for automatic multilevel thresholding. *IEEE Transactions on Image Processing* 4, 370-378 (1995).

3 Zack, G. W., Rogers, W. E. & Latt, S. A. Automatic measurement of sister chromatid exchange frequency. *Journal of Histochemistry & Cytochemistry* 25, 741-753 (1977).

4 Prewitt, J. & Mendelsohn, M. The analysis of cell images. *Annals of the New York Academy of Sciences* 128, 1035-1053 (1966).

5 Carpenter, A. E. *et al.* CellProfiler: image analysis software for identifying and quantifying cell phenotypes. *Genome Biol* 7, R100; 10.1186/gb-2006-7-10-r100 (2006).

6 Valente, M., Voisin, P., Laloi, P., Roy, L. & Roch-Lefèvre, S. Automated gamma-H2AX focus scoring method for human lymphocytes after ionizing radiation exposure. *Radiation Measurements* 46, 871-876; 10.1016/j.radmeas.2011.05.012 (2011).

7 Runge, R. *et al.* Fully automated interpretation of ionizing radiation-induced γH2AX foci by the novel pattern recognition system AKLIDES®. *Int J Radiat Biol* 88, 439-447; 10.3109/09553002.2012.658468 (2012).

8 Willitzki, A. *et al.* Fully automated analysis of chemically induced gammaH2AX foci in human peripheral blood mononuclear cells by indirect immunofluorescence. *Cytometry A* 83, 1017-1026; 10.1002/cyto.a.22350 (2013).

9 Qvarnström, O. F., Simonsson, M., Johansson, K.-A., Nyman, J. & Turesson, I. DNA double strand break quantification in skin biopsies. *Radiother Oncol* 72, 311-317; 10.1016/j.radonc.2004.07.009 (2004).

10 Böcker, W. & Iliakis, G. Computational Methods for analysis of foci: validation for radiation-induced gamma-H2AX foci in human cells. *Radiat Res* 165, 113-124 (2006).

11 Ivashkevich, A. N. *et al.* γH2AX foci as a measure of DNA damage: a computational approach to automatic analysis. *Mut Res* 711, 49-60; 10.1016/j.mrfmmm.2010.12.015 (2011).

12 Jakl, L. *et al.* Validation of JCountPro software for efficient assessment of ionizing radiation-induced foci in human lymphocytes. *Int J Radiat Biol* 92, 766-773; 10.1080/09553002.2016.1222093 (2016).

13 Herbert, A. D., Carr, A. M. & Hoffmann, E. FindFoci: A Focus Detection Algorithm with Automated Parameter Training That Closely Matches Human Assignments, Reduces Human Inconsistencies and Increases Speed of Analysis. *PloS One* 9, e114749; 10.1371/journal.pone.0114749 (2014).
